# Combination of red blood cell distribution width and body mass index (COR-BMI) predicts in-hospital mortality in patients with different diagnoses?

**DOI:** 10.1371/journal.pone.0219549

**Published:** 2019-07-15

**Authors:** Isabela Borges Ferreira, Emanuelle do Nascimento Santos Lima, Nayara Cristina da Silva, Isaías Valente Prestes, Geórgia das Graças Pena

**Affiliations:** 1 Multiprofessional Residence Program, Federal University of Uberlandia, Uberlandia, Minas Gerais, Brazil; 2 Graduate Program in Health Sciences, Federal University of Uberlandia, Uberlandia, Minas Gerais, Brazil; 3 Federal University of Rio Grande do Sul, Porto Alegre, Rio Grande do Sul, Brazil; Universidad Miguel Hernandez de Elche, SPAIN

## Abstract

**Background:**

The combination of red blood cell distribution width and body mass index (COR-BMI) is indicated as a new prognostic index of survival in patients with laryngeal cancer. However, the ability of this prediction in other types of cancer or whether its use can be expanded to non-oncological patients is unknown. The aim of this study was to investigate the prediction of prognosis of in-hospital mortality of the COR-BMI in oncological and non-oncological patients.

**Methods:**

A retrospective study was performed with all hospitalized patients between 2014 and 2016, totaling 2930 patients, 262 oncological and 2668 non-oncological. The COR-BMI was divided into three classes: 0, RDW ≤ 13.1% and BMI ≥ 25 kg/m^2^; 1, RDW ≤ 13.1% and BMI < 18.5 or ≥ 18.5 but < 25 kg/m^2^ and RDW > 13.1% and BMI ≥ 18.5 but < 25 or BMI ≥ 25 kg/m^2^; and 2, RDW > 13.1% and BMI < 18.5 kg/m^2^. In order to analyze the relationship between COR-BMI and in-hospital mortality in the studied population, the Cox Proportional Hazards Model was used in a multivariate analysis based on a conceptual model.

**Results:**

The COR-BMI was an independent predictor of in-hospital mortality in non-oncological patients (1 versus 0: HR = 3.34; CI = 1.60–6.96, p = 0.001; 2 versus 0: HR = 3.38; CI = 1.22–9.39, p = 0.019). The survival rate of these patients was lower among those with the highest scores on the COR-BMI. This prediction was not found in oncological patients.

**Conclusion:**

The present study suggests that the COR-BMI may have its practical use expanded to non-oncological patients as an independent predictor of in-hospital mortality.

## Introduction

Hospitalized patients have a high mortality rate [1]. Risk factors and prognoses related to mortality from certain diseases are not even fully understood [1, 2]. Several mechanisms have been associated with the organic variations that occur due to the advent or evolution of the disease. If previously noted and monitored, these variations may result in better survival [3].

The red blood cell distribution width (RDW) is an indicator that represents impaired erythropoiesis [4]. It is used for the differential diagnosis of anemia; however, anisocytosis is also related to several acute or chronic diseases [5]. In addition, a high RDW is considered an independent risk factor for mortality in the general population [4].

High values of RDW are related to lower survival [6], longer length of hospital stay, and in-hospital mortality [7]. In addition, it is suggested that its elevation also presents a prognostic value of lower survival in oncological patients [8, 9]. This was observed in patients with gastric [10], lung [6], renal [11], and hematological neoplasias [12].

One explanation for such a situation would be that high values of RDW may be associated with inflammation, oxidative stress, and changes in erythropoiesis as well as other conditions that negatively impact mortality [5]. The mechanism of inflammation in RDW values may exist due to the production of inflammatory markers that hinder the response to erythropoietin and reduce erythrocyte survival [13].

Systemic inflammation is associated with a decrease in total body mass, especially lean mass [14]. Studies report that a body mass index (BMI) below the recommendation was related to a worse prognosis and to postoperative complications [15], significantly increasing the in-hospital mortality rate [16].

Although it is not considered an ideal index when used separately, BMI is still being widely used, because it has a validated outcome predictive value. Because of this fact, the European Society for Clinical Nutrition and Metabolism (ESPEN), in order to supply a set of consensus-based criteria for diagnosing malnutrition, considered that the BMI < 18.5 kg/m^2^, in patients undergoing screening and classified as at risk of malnutrition, is an efficient alternative to perform this evaluation [17].

The BMI is also used in studies that evaluate the obesity paradox; in other words, the observation that patients classified as overweight and obese have lower mortality. These classifications are then considered as protective against some conditions or diseases [18, 19]. According to the Wang et al. [20] meta-analysis, patients classified as overweight by BMI showed a reduction in in-hospital mortality when compared to eutrophic patients.

In this sense, a new prognostic index of survival of oncological patients was described in the literature. Proposed by Fu et al. [21], this index considered inflammation and nutrition by the combination of red blood cell distribution width and body mass index (COR-BMI). It was evaluated in patients with a diagnosis of laryngeal squamous cell carcinoma and showed that those with the highest scores on the COR-BMI represented the worst prognosis. In addition, the COR-BMI was considered an independent predictor of cancer-specific survival [21]. However, the ability of this prediction in other types of cancer or whether its use can be expanded to non-oncological patients is unknown.

Thus, the aim of this study was to investigate the prediction capacity of prognosis of in-hospital mortality of the COR-BMI in oncological and non-oncological patients.

## Materials and methods

### Ethical aspects

This study was approved by the Human Research Ethics Committee of the Federal University of Uberlandia by protocol CAAE n° 65340116.8.0000.5152. All data were fully anonymized before you accessed them and ethics committee agreed to waive the requirement for informed consent.

### Population and study design

**Fig 1:**
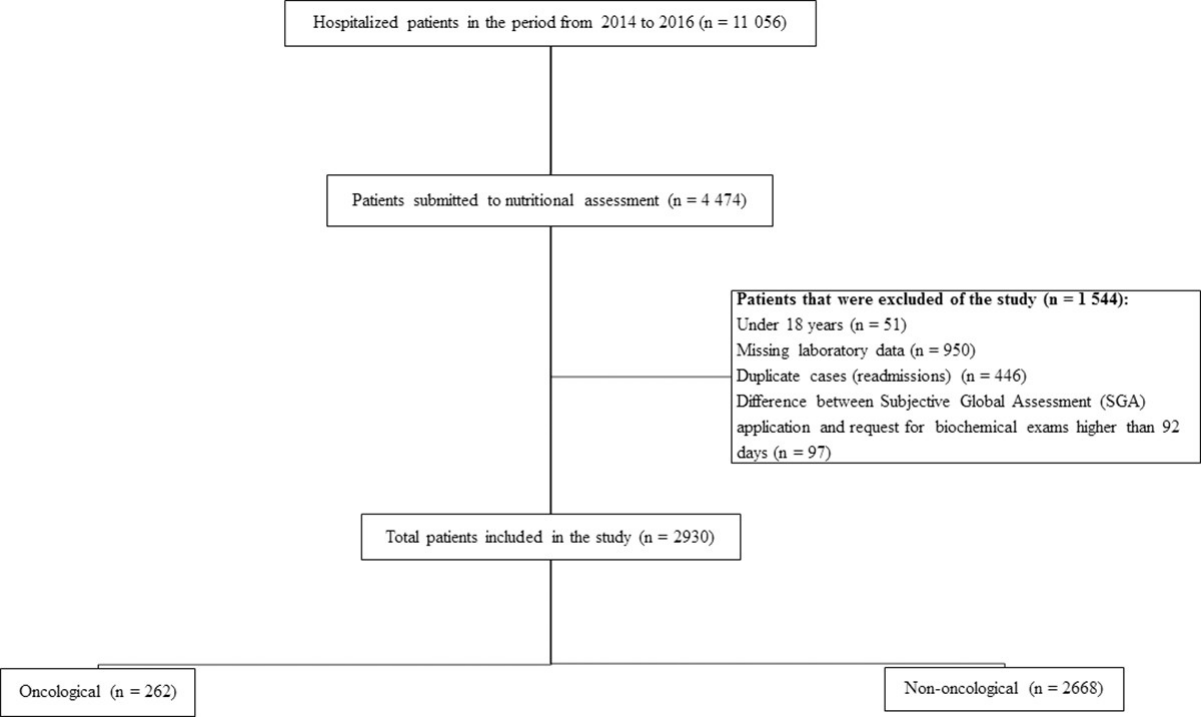
Diagram describing the process of selection of the study participants.

A retrospective study with all hospitalized patients from January 1, 2014 to December 31, 2016 was performed at the following hospitalization units: Internal Medicine, Surgical I (Traumatology, Neurology and Urology), Surgical II (Thoracic, Gastrointestinal Tract and General), Surgical III (Vascular and General), Coronary Unit, Thoracic Pain Unit, Infectious Diseases, Adult Emergency Room and Emergency Room. This study included 2930 patients, 262 oncological and 2668 non-oncological belonging to tertiary hospital (Fig 1).

### Eligibility criteria

All patients 18 years or older who underwent nutritional assessment and presented laboratory data with RDW results participated in this study (Fig 1).

### Data collection

Demographic information, such as age group (years) and gender (female/male), and clinic information, such as diagnosis, tumor site, ward, date and length of stay (days), BMI (kg/m^2^), RDW (%), nutritional diagnosis from Subjective Global Assessment (SGA)—Well-nourished (A), Moderately malnourished (B), Severely malnourished (C) -, hospital discharge and death, were extracted from the Hospital Information System (HIS), retrospectively.

The first SGA performed after hospitalization and the RDW that was collected in the first request of patient exams, with the majority of the results obtained on the first day of hospitalization, were considered. The maximum difference between the date of hospitalization and the request for RDW was 22 days in a single patient, and for 13 patients, the maximum was approximately 10 days.

### Determination of RDW, BMI and COR-BMI cut-off values

For the determination of the RDW, BMI and COR-BMI cut-off values, the classifications of the original study were followed. The RDW was divided into two classes, ≤ 13.1% and > 13.1%, and the BMI into three classes, < 18.5 kg/m^2^; ≥ 18.5 but < 25 kg/m^2^; and ≥ 25 kg/m^2^. For the classification of COR-BMI, three categories were used: 0, RDW ≤ 13.1% and BMI ≥ 25 kg/m^2^; 1, RDW ≤ 13.1% and BMI < 18.5 kg/m^2^ or ≥ 18.5 but < 25 kg/m^2^ and patients with RDW > 13.1% and BMI ≥ 18.5 but < 25 kg/m^2^ or BMI ≥ 25 kg/m^2^; and 2, RDW > 13.1% and BMI < 18.5 kg/m^2^ [21].

To know if our distribution was approximated from the original study, a cut-off of 13.5 for the RDW were delimited by the Receiver Operating Characteristic (ROC) (sensitivity 64.9%, specificity 61.3%, Area Under the Curve [AUC] 0.63, 95% CI 0.61–0.65).

In order to observe the statistical power to the oncological group, we performed the post hoc considering an odds ratio of 3.0 (COR-BMI 0 *versus* 1 + 2), the sample size of 245, we have obtained the sample power of 0.99. This statistical was performed in G*Power Software, version 3.1.

### Confounding variables

Sex and age group were considered potential confounding variables in the present study.

### Statistical analysis

The descriptive statistics percentage, mean, median, and standard deviation were used to describe the population according to the classifications in the COR-BMI and clinical outcomes (hospital discharge and death). The differences between the three scoring groups for COR-BMI and between the patients who were discharged from the hospital and who died were assessed using the Pearson Chi-Square Test for categorical variables and the Analysis of Variance (ANOVA) for continuous variables.

In order to analyze the relationship between the COR-BMI and in-hospital mortality in the studied population, univariate and multivariate regression analyses were used, using the Cox Proportional Hazards Model, based on a conceptual model. In order to identify the probability of in-hospital mortality according to the Kaplan-Meier method, survival curves were used, and the differences between the curves were compared from the Log-Rank Test.

A confidence interval (CI) of 95% and p value < 0.05 were considered as the levels of statistical significance. The data were compiled and analyzed using STATA software version 12.0 (STATA Corporation, College Station, TX, USA). For analysis of survival curves, the Statistical Package for Social Sciences (SPSS) version 20.0 (SPSS Inc., Chicago, IL, USA) was used.

## Results

This study included 262 oncological patients with a mean age of 62.2 ± 13.6 years, mostly male (50.6%) and elderly (63.3%), and 2668 non-oncological patients with a mean age of 55.4 ± 17.6 years, predominantly male (60.0%) and adults (55.6%) (Table 1).

**Table 1:** Clinical and nutritional characteristics of oncological and non-oncological patients according to COR-BMI ratings.

Oncological Non-oncological								
Variables	COR- BMI 0	COR- BMI 1	COR- BMI 2	P value^c^	COR- BMI 0	COR- BMI 1	COR- BMI 2	P value^c^
	N (%)	N (%)	N (%)		N (%)	N (%)	N (%)	
**Age group (years)** <60 ≥60	19 (43.2) 25 (56.8)	66 (36.1) 117 (63.9)	5 (27.8) 13 (72.2)	0.486	403 (60.2) 266 (39.8)	862 (54.2) 728 (45.8)	56 (48.7) 59 (51.3)	0.010
**Sex** Female Male	19 (43.2) 25 (56.8)	95 (51.9) 88 (48.1)	7 (38.9) 11 (61.1)	0.379	260 (38.9) 409 (61.1)	646 (40.6) 944 (59.4)	44 (38.3) 71 (61.7)	0.682
**Tumor site** GI FGS MGS Metastasis HN Others^a^	15 (34.1) 2 (4.5) 6 (13.6) 9 (20.4) 2 (4.5) 10 (22.8)	60 (32.8) 6 (3.3) 18 (9.8) 69 (37.7) 8 (4.4) 22 (11.9)	7 (38.9) 0 (0.0) 1 (5.6) 10 (55.6) 0 (0.0) 0 (0.0)	0.154	-	-	-	-
**Wards** Internal Medicine Surgical I Surgical II Surgical III Others^b^	5 (11.4) 5 (11.4) 31 (70.5) 2 (4.5) 1 (2.3)	24 (13.1) 6 (3.3) 124 (67.8) 17 (9.3) 12 (6.6)	2 (11.1) 0 (0.0) 13 (72.2) 3 (16.7) 0 (0.0)	0.222	175 (26.2) 102 (15.2) 151 (22.6) 91 (13.6) 150 (22.4)	484 (30.4) 148 (9.3)* 437 (27.5) 190 (11.9) 331 (20.8)	34 (29.6) 6 (5.2)* 31 (27.0) 15 (13.0) 29 (25.2)	0.001
**Length of stay (days)** <11 ≥11	27 (61.4) 17 (38.6)	102 (55.7) 81 (44.3)	11 (61.1) 7 (38.9)	0.747	386 (57.7) 283 (42.3)	732 (46.0) 858 (54.0)	40 (34.8) 75 (65.2)	0.001
**Nutritional Diagnosis** Well-nourished Mildly-malnourished Severely malnourished	32 (72.7)** 10 (22.7)** 2 (4.5)**	43 (23.5)** 95 (51.9)** 45 (24.6)	0 (0.0)** 6 (33.3) 12 (66.7)**	0.001	447 (68.7)** 184 (28.3)** 20 (3.1)**	661 (42.0)** 688 (43.7)** 225 (14.3)	2 (1.7)** 49 (42.6) 64 (55.6)**	0.001

Abbreviations: COR-BMI = combination of red blood cell distribution width and body mass index; GI = gastrointestinal tract; FGS = female genital system; MGS = male genital system; HN = head and neck

^a^ = breast, urinary tract, trachea, hematological, lung, skin and osteosarcoma; Surgical I = Traumatology, Neurology and Urology; Surgical II = Thoracic, Gastrointestinal Tract and General; Surgical III = Vascular and General

^b^ = Coronary Unit, Thoracic Pain Unit, Infectious Diseases, Adult Emergency Room and Emergency Room. n may vary according to the variability of the data.

^c^ Pearson Chi-square test.

*P-value Bonferroni test ≤0.003.

**P-value Bonferroni test ≤0.005.

Regarding the distribution of these variables into the three COR-BMI classes for oncological patients, 44 (18.0%) had COR-BMI 0, 183 (74.7%) had COR-BMI 1 and 18 (7.3%) COR-BMI 2, whereas in the non-oncological patients, 669 (28.2%) had COR-BMI 0, 1590 (67.0%) had COR-BMI 1 and 115 (4.8%) COR-BMI 2.

The majority of oncological patients presented, respectively, metastasis (35.9%), tumor into the gastrointestinal tract (33.5%) and male genitalia (10.2%).

According to Table 1, only malnutrition (p = 0.001) was associated with the COR-BMI in oncological patients, whereas in non-oncological patients, the age group over 60 years (p = 0.010), the Surgical I (p = 0.001), longer length of stay (p = 0.001) and malnutrition (p = 0.001) were associated. In addition, the higher frequency of oncological and non-oncological patients with the COR-BMI 2 were diagnosed with severe malnutrition according to SGA.

Regarding the analysis of hospital discharge and death (Table 2), 28 (10.7%) oncological patients and 109 (4.1%) non-oncological patients died during hospitalization, and the median length of stay was 11 days (1–324 days). In relation to the nutritional diagnosis by SGA, 181 (69.1%) oncological patients and 1428 (54.3%) non-oncological patients were diagnosed with moderate or severe malnutrition.

**Table 2:** Association between the variables, hospital discharge and death among the oncological and non-oncological patients.

Oncological Non-oncological						
Variables	Hospital Discharge	Death	P value^c^	Hospital Discharge	Death	P value^c^
	N (%)	N (%)		N (%)	N (%)	
**Age group (years)** <60 ≥60	88 (37.6) 146 (62.4)	10 (35.7) 18 (64.3)	0.845	1454 (56.8) 1105 (43.2)	27 (24.8) 82 (75.2)	0.001
**Sex** Female Male	114 (48.7) 120 (51.3)	16 (57.1) 12 (42.9)	0.399	1044 (40.8) 1515 (59.2)	37 (33.9) 72 (66.1)	0.154
**Tumor site** GI FGS MGS Metastasis HN Others^a^	76 (32.5) 8 (3.4) 26 (11.1) 80 (34.2) 12 (5.1) 32 (13.6)	9 (32.1) 0 (0.0) 3 (10.7) 14 (50.0) 0 (0.0) 2 (7.2)	0.969	-	-	-
**Wards** Internal Medicine Surgical I Surgical II Surgical III Others^b^	31 (13.2) 13 (5.6) 158 (67.5) 17 (7.3) 15 (6.4)	4 (14.3) 0 (0.0) 16 (57.1) 6 (21.4) 2 (7.1)	0.103	716 (28.0) 308 (12.0) 624 (24.4) 316 (12.3) 595 (23.3)	25 (22.9) 10 (9.2) 34 (31.2) 13 (11.9) 27 (24.8)	0.440
**Length of stay (days)** <11 ≥11	137 (58.5) 97 (41.4)	13 (46.4) 15 (53.6)	0.221	1260 (49.2) 1299 (50.8)	21 (19.3) 88 (80.7)	0.001
**Nutritional Diagnosis** Well-nourished Mildly-malnourished Severely malnourished	79 (33.8)* 103 (44.0) 52 (22.2)*	2 (7.1)* 13 (46.4) 13 (46.4)*	0.003	1184 (46.9)* 988 (39.2)* 348 (13.8)*	15 (14.0)* 58 (54.2)* 34 (31.8)*	0.001
**COR-BMI** 0 1 2	43 (19.5) 163 (74.1) 14 (6.4)	1 (4.0) 20 (80.0) 4 (16.0)	0.051	661 (28.9)* 1517 (66.4)* 108 (4.7)	8 (9.1)* 73 (82.9)* 7 (7.9)	0.001

Abbreviations: GI = gastrointestinal tract; FGS = female genital system; MGS = male genital system; HN = head and neck

^a^ = breast, urinary tract, trachea, hematological, lung, skin and osteosarcoma; Surgical I = Traumatology, Neurology and Urology; Surgical II = Thoracic, Gastrointestinal Tract and General; Surgical III = Vascular and General

^b^ = Coronary Unit, Thoracic Pain Unit, Infectious Diseases, Adult Emergency Room and Emergency Room; COR-BMI = combination of red blood cell distribution width and body mass index. n may vary according to the variability of the data.

^c^ Pearson Chi-square test.

*P-value Bonferroni test ≤0.008.

In the oncological patients, just malnutrition (p = 0.003) were associated with the clinical outcome of death, whereas in the non-oncological patients, age group over 60 years (p = 0.001), longer length of stay (p = 0.001), malnutrition (p = 0.001) and higher scores on the COR-BMI index (p = 0.001) were associated with the clinical outcome (Table 2).

According to Table 3, in the univariate analysis, age group over 60 years (p = 0.001) and higher score on the COR-BMI index (p = 0.008) increased the risk of in-hospital mortality in the non-oncological patients and, even when adjusted for other variables using the multivariate analysis of the Cox Proportional Hazards Model, the highest COR-BMI scores increased the chance of in-hospital mortality by 3.4 times (1 versus 0: HR = 3.34; CI = 1.60–6.96, p = 0.001; 2 versus 0: HR = 3.38; CI = 1.22–9.39, p = 0.019). In the oncological patients, this association was not found.

**Table 3:** Cox Regression Analysis for in-hospital mortality in oncological and non-oncological patients.

Oncological Non-oncological								
Variables	Univariate	P value^c^	Multivariate	P value^c^	Univariate	P value^c^	Multivariate	P value^c^
	HR (95% CI)		HR (95% CI)		HR (95% CI)		HR (95% CI)	
**Age group** <60 ≥60	1 (reference) 0.81 (0.37–1.78)	0.608	1 (reference) 1.28 (0.52–3.16)	0.594	1 (reference) 3.96 (2.56–6.11)	0.001	1 (reference) 4.68 (2.78–7.87)	0.001
**Sex** Female Male	1 (reference) 0.59 (0.27–1.28)	0.184	1 (reference) 0.58 (0.25–1.34)	0.201	1 (reference) 1.06 (0.71–1.58)	0.772	1 (reference) 1.35 (0.85–2.14)	0.208
**COR-BMI** 0 1 2	1 (reference) 3.86 (0.51–28.93) 7.06 (0.78–63.52)	0.189 0.081	1 (reference) 3.83 (0.51–28.76) 7.26 (0.81–65.43)	0.192 0.077	1 (reference) 3.23 (1.55–6.69) 3.96 (1.43–10.95)	0.002 0.008	1 (reference) 3.34 (1.60–6.96) 3.38 (1.22–9.39)	0.001 0.019

Abbreviations: COR-BMI = combination of red blood cell distribution width and body mass index. n may vary according to the variability of the data. HR = Hazard-ratio.

^c^ Cox Regression.

**Fig 2:**
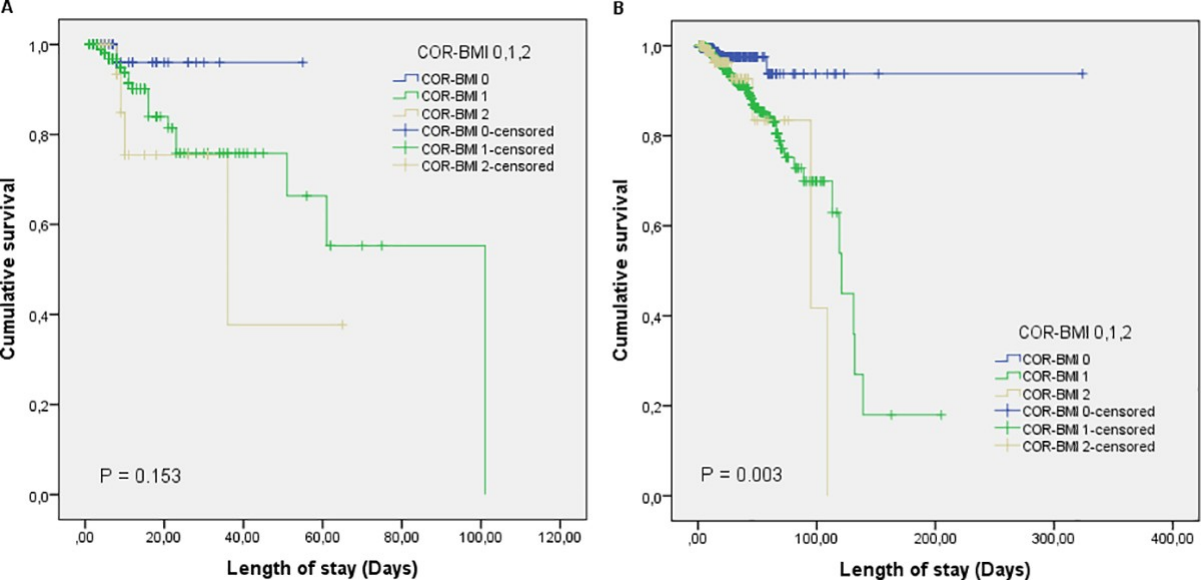
Kaplan-Meier curves for the survival rates. (A) Kaplan-Meier curves for the survival of the oncological patients categorized by COR-BMI score 0,1 and 2. (B) Kaplan-Meier curves for the survival of the non-oncological patients categorized by COR-BMI score 0,1 and 2. Abbreviations: COR-BMI = combination of red blood cell distribution width and body mass index.

In Fig 2B, we observed that the survival rate of non-oncological patients with COR-BMI 2 was significantly lower than that of patients with COR-BMI 1 and 0; this difference was significant according to the Log-Rank Test (p = 0.003). However, this prediction was not found in the oncological patients (Fig 2A).

## Discussion

In this study, we investigated the association of the COR-BMI with in-hospital mortality in oncological and non-oncological patients. The COR-BMI increased the chance of in-hospital mortality in non-oncological patients by more than 3 times. From our knowledge, this is the first study to show this relationship in non-oncological patients.

Furthermore, the survival rate of non-oncological patients with higher scores on the COR-BMI was shorter when compared to those with lower scores. This prediction was not verified in oncological patients, but the discovered association was different from that reported in the original study [21] in which the cancer-specific survival was evaluated in long-term and, in this study, in-hospital mortality was analyzed in short-term.

Therefore, the research with this new indicator was restricted to oncological patients. In this study, the COR-BMI function was observed for the non-oncological patients, different from that found by Fu et al. [21] in patients with laryngeal squamous cell carcinoma. One explanation for not having an association between the COR-BMI and in-hospital mortality in oncological patients would be the short length of hospital stay.

As this indicator derives from the RDW and BMI values, they cannot be left behind in the discussion. The RDW was cited in several studies as a predictor of mortality [22–24]. Beyond mortality, it was also cited as a predictor of rehospitalization in patients with chronic heart failure [25] or death due to coronary artery disease [22].

On the other hand, low BMI is associated with worse survival. It was considered an independent prognostic factor for survival in gastric cancer patients who underwent gastrectomy. These patients classified as having low BMI presented an increased risk of mortality when compared to the other classifications [15]. Studies indicate that malnutrition is related to increased mortality, decreased quality of life, long hospital stay, reduced tolerance to treatment, and shortened survival [26–28].

In a multinational cohort, Cereda et al. [29] analyzed the association between BMI and age with in-hospital mortality of 97.344 adult patients and found that both were independent predictors of in-hospital mortality. Besides that, the classification of underweight performed by BMI was considered a risk factor for mortality, and the classifications of overweight and obesity might confer a protective effect. This situation refers to the concept of the obesity paradox.

This concept was also supported by the findings of Yamauchi et al. [30] with 263.940 elderly patients hospitalized with chronic obstructive pulmonary disease, where those classified as being overweight and obese by BMI had lower mortality. These considerations highlight the importance of the routine evaluation of the nutritional status of hospitalized patients.

In this sense, the SGA is a useful tool to evaluate the nutritional diagnosis of surgical and hospitalized patients [31]. In a prospective study performed with 200 adult patients admitted to the intensive care unit (ICU), Verghese et al. [32] found that 48.5% had moderate malnutrition and 6.5% severe malnutrition, according to the assessment made by SGA. It was also observed that patients classified with some degree of malnutrition had a greater chance of mortality. Similar results were found by Lew et al. [33] in a prospective cohort performed with 439 patients admitted to the ICU, where those who had malnutrition also had a higher mortality risk.

In the study of Konturek et al. [34] with 815 hospitalized patients, it was found 53.6% of malnutrition in patients assessed by SGA and 44.6% in nutritional risk according to the Nutritional Risk Screening (NRS 2002); however, malnutrition was not recorded by 84.5% of doctors. This shows that many times the nutritional status is not valued in clinical practice or at least not registered in an appropriate way. Is widely known that BMI is associated to a worse prognosis, clinical complications and mortality [15, 16]. In addition, a higher RDW also is considered an independent risk factor for mortality in a miscellaneous clinical conditions [6–12]. Thus, a simple indicator that assesses inflammation and nutrition concomitantly can be promising.

In addition, the majority of the oncological and non-oncological patients who scored higher on the COR-BMI were diagnosed with severe malnutrition, which suggests a possible relationship between the COR-BMI scores and nutritional status. This relationship is also suggested in the study by Fu et al. [21], however, according to the reduction of hemoglobin levels and increase in the COR-BMI scores. Thus, with this single indicator, we could evaluate important factors that, independently, have demonstrated its potential prognosis in the literature.

The COR-BMI was considered an independent predictor of cancer-specific survival [21]. In the study by Souza et al. [35] conducted with oncological patients in palliative care, malnutrition was evaluated by the scored Patient-Generated Subjective Global Assessment (PG-SGA) and the intensity of systemic inflammation by the modified Glasgow Prognostic Score (mGPS), which found that both were independent predictors of survival in the studied population. For the classification of the COR-BMI, only RDW and BMI values are necessary. So, it is practical to use it as a prognostic factor, as it can be routinely used in all hospitalized patients.

This study has some limitations. Firstly, we could not have enough oncological patients to perform a more specific analysis with this group because they were just hospitalized to surgical procedures and did not continue the treatment in the included wards. Besides that, the oncological patients had various types of cancer and different times of diagnosis in the sample. This variability could had influenced these results since we not observed significance to oncological patients, although all hospitalized in the period were considered. Secondly, the retrospective study could show some bias, such as the inaccuracy or missing of data; e.g., the anthropometric measures made by different health professionals or lack of requested of biochemical exams. Moreover, it is necessary to analyze the different cut-off points for RDW present in the literature. Because of that, we performed the ROC curve founding a cut-off point adequate for this population. Finally, we did not consider the vitamin B12 or folate exams because they are not routinely available at the hospital that could, ultimately, affect RDW levels.

As positive aspects, this study presents, for the first time, the advantage of using a simple index that assists in the prediction of short-term mortality prognosis in hospitalized non-oncological patients with different diagnoses. Besides, it suggests a possible relationship between this index and nutritional status. Thus, it is possible to guide a higher level of nutritional attention and to minimize the underreporting of malnutrition.

At last, more studies are necessary to understand the performance of the COR-BMI in primary and/or secondary levels of health care since we have already been found in non-oncological patients with different diagnoses. Besides that, studies with a larger number of participants are needed to evaluate the prediction of prognosis by the COR-BMI in patients with different types of cancer.

## Conclusion

The present study suggests that the COR-BMI may have its practical use expanded to non-oncological patients as an independent predictor of in-hospital mortality.

## Supporting information

S1 File The database file for this manuscript (Microsoft Excel format). (XLS)
